# 779. PIDS Featured Oral Abstract: High Beta Lactam Exposure in Neonates with Bloodstream Infection Associated with Intestinal Dysbiosis and *Enterococcus spp.* Abundance in the Gut Microbiome

**DOI:** 10.1093/ofid/ofac492.041

**Published:** 2022-12-15

**Authors:** Hope Hendricks, Jörn-Hendrik Weitkamp, Suman Pakala, Seesandra Rajagopala, Ritu Banerjee

**Affiliations:** Vanderbilt University Medical Center, Nashville, TN; Vanderbilt University Medical Center, Nashville, Tennessee; Vanderbilt University Medical Center, Nashville, TN; Vanderbilt University Medical Center, Nashville, TN; Vanderbilt University Medical Center, Nashville, TN

## Abstract

**Background:**

To characterize how antibiotic exposure impacts development and durability of intestinal dysbiosis and the acquisition of antibiotic resistance genes (ARGs) in the intestinal flora of premature infants in the Neonatal Intensive Care Unit (NICU), we established an infant gut microbiome biorepository (IGMB).

**Methods:**

We performed prospective weekly stool collection in NICU patients meeting the following criteria: birthweight < 2000 g, postnatal age < 2 months and no diagnoses of congenital gut malformation or cyanotic heart disease. Cases were infants with bloodstream infections (BSI), defined as bacterial growth from blood culture; controls were infants with < 5 days of antibiotic exposure, no BSI nor necrotizing enterocolitis. We performed metagenomic analysis on 5–6 serial stool samples from each of the 10 cases and 10 controls (n= 100 stools). We used Wilcoxon rank sum tests for pairwise comparisons.

**Results:**

From July 2021 to May 2022, 265 infants contributed 1,300 stool samples to the IGMB. In 7 of 8 BSI cases the causative pathogen was identified in the pre-BSI stool sample. Two more BSI cases did not have a pre-BSI stool sample. Microbiome species α-diversity increased with advancing postnatal age in controls but not in cases (Fig. 1). Among 6 cases with high beta lactam exposure ( >14 cumulative days by last stool collection), 3 had notable increases in the relative abundance of *Enterococcus spp*. (Fig. 2). Controls did not have similar trends in *Enterococcus spp*., but did have higher relative abundance of facultative anaerobes including *Bifidobacterium, Lactobacillus*, and *Veillonella spp*. (Fig. 3). Antibiotic resistance gene (ARG) abundance did not differ significantly between cases and controls.

**Conclusion:**

Compared to controls, neonates with BSI have increased antibiotic intensity throughout their NICU admissions, reduction of microbial species diversity and overabundance of specific organisms in their gut microbiomes. We observed increased *Enterococcus spp.* prevalence with higher beta lactam exposure. ARG abundance did not differ between cases and controls. The clinical implications of this microbiome dysbiosis warrant further study.

**Disclosures:**

**Jörn-Hendrik Weitkamp, MD**, Roche Diagnostics: Advisor/Consultant.

**Figure 1:**
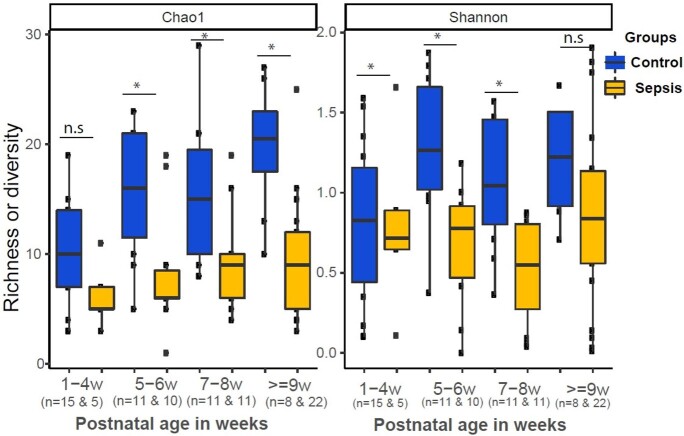
Diversity indices by postnatal age for the prospective, longitudinal stool samples of 10 cases and 10 controls. The * indicates statistically significant differences between cases and controls (p<0.05) and n.s., non-significant.

**Figure 2:**
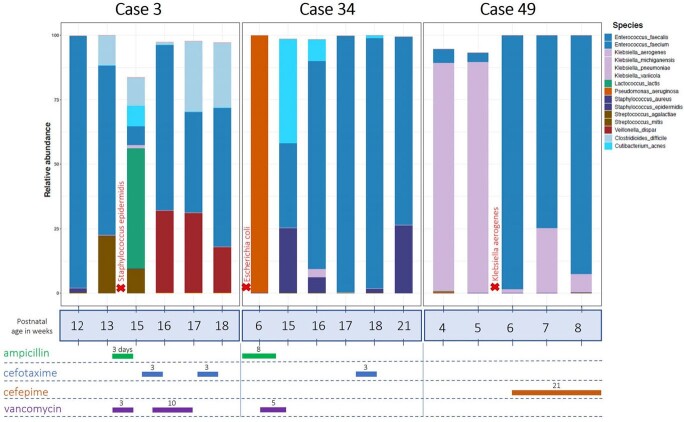
Longitudinal stool samples for three patients with BSI and high beta lactam exposure (prior to the first stool sample, cumulative beta lactam days of therapy were 34, 42, and 11 days for cases 3, 34, and 49, respectively). BSI timing and pathogen are indicated by red X. Relative abundances both before and after the BSI show low overall species diversity, few commensal organisms, and an overabundance of Enterococcus spp.

**Figure 3:**
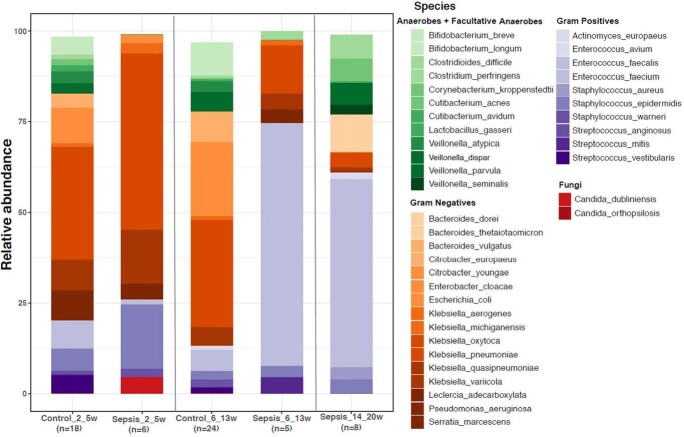
Stool samples are grouped by advancing postnatal age (2–5, 6–13 and 14–20 weeks) for BSI cases and controls; the top 40 most abundant organisms identified by metagenomic analysis are shown. Controls have a higher abundance and variety of anaerobes and facultative anaerobes than cases. Cases develop a high percentage of Enterococcus spp. at 6–13 weeks that persists through 14–20 weeks.

